# Supplementary material for: GAL4-based functional screen of neuropeptides in Drosophila reproduction
Source: PLoS One. 2026 Mar 27;21(3):e0345918. doi: 10.1371/journal.pone.0345918 (PMC13028521; doi:10.1371/journal.pone.0345918)
Supplement: S1 File — Representative whole-mount ovary images showing NP-GAL4 > UAS-mCD8::GFP expression, immunostained with anti-GFP (green), DAPI (blue), phalloidin (red). (PDF) [file pone.0345918.s001.pdf]

NP-GAL4>UAS-mCD8::GFP expression in the ovaries

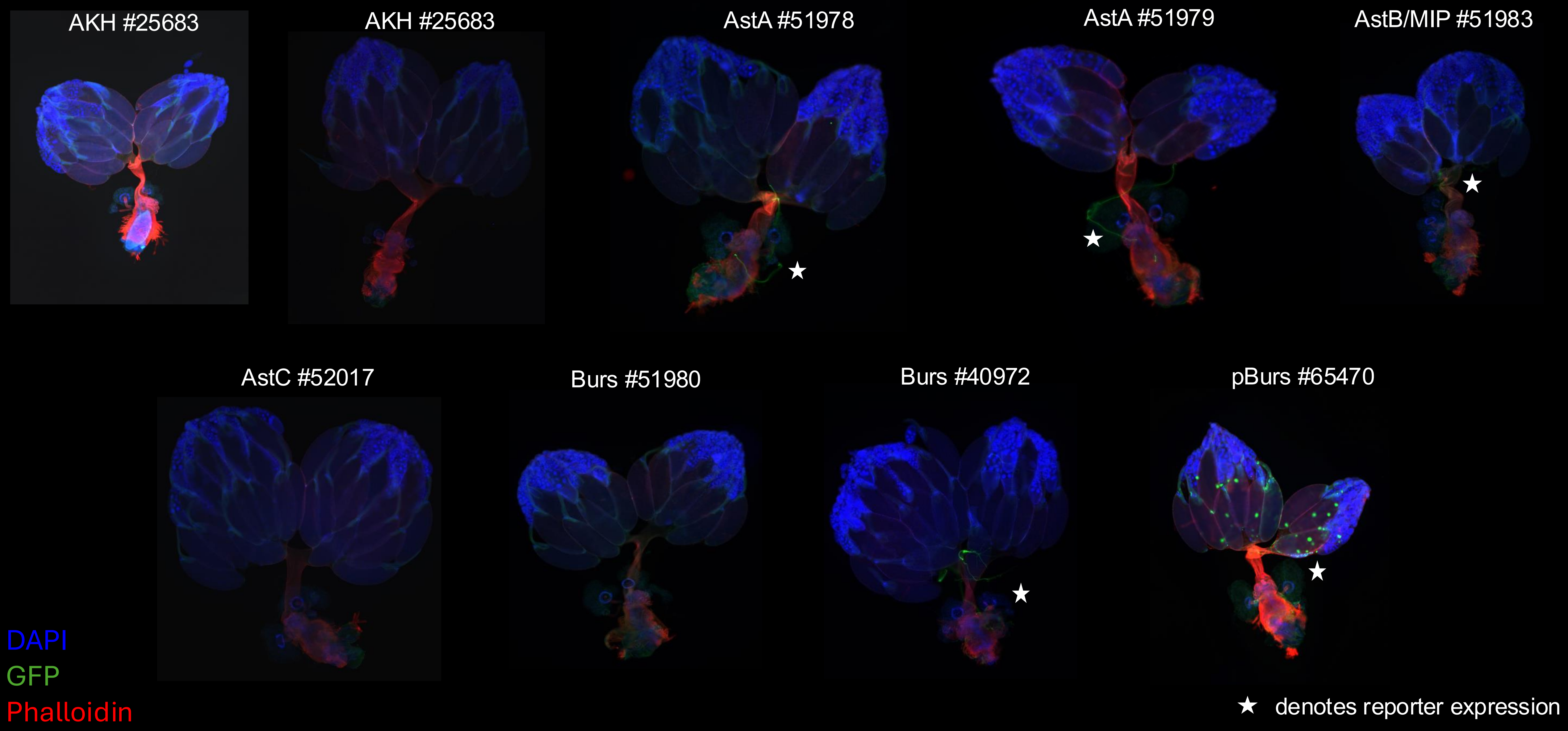

NP-GAL4>UAS-mCD8::GFP expression in the ovaries

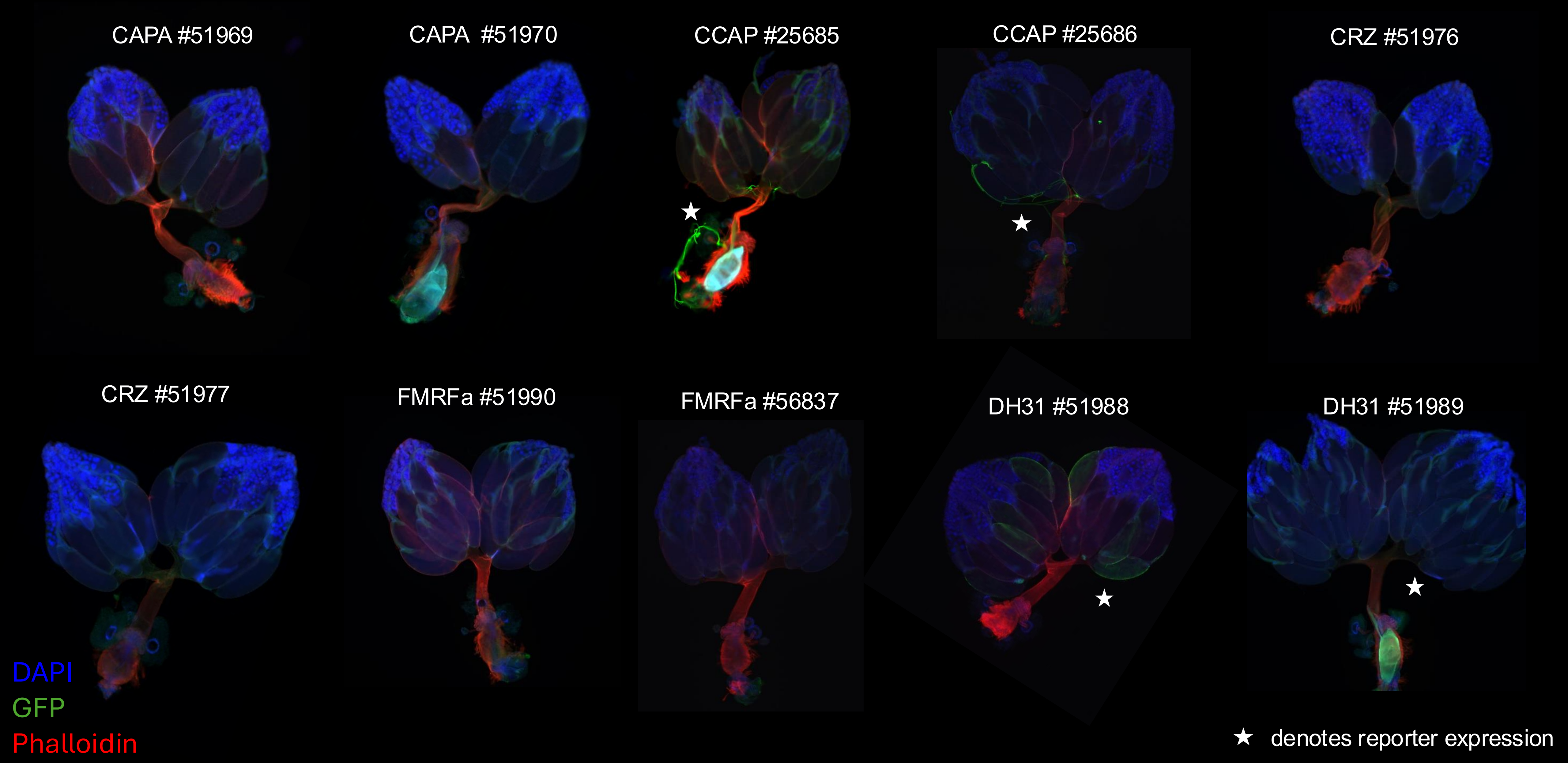

NP-GAL4>UAS-mCD8::GFP expression in the ovaries

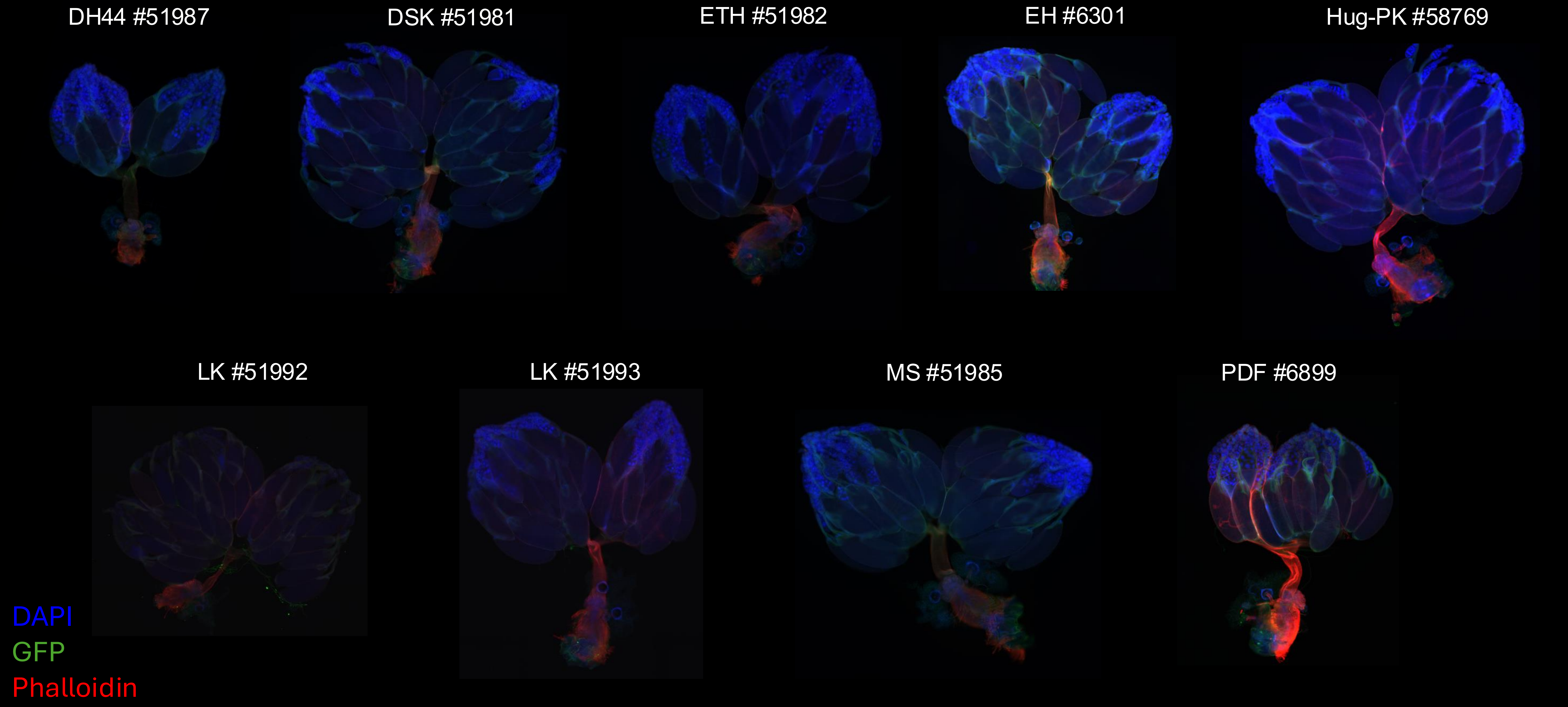

NP-GAL4>UAS-mCD8::GFP expression in the ovaries

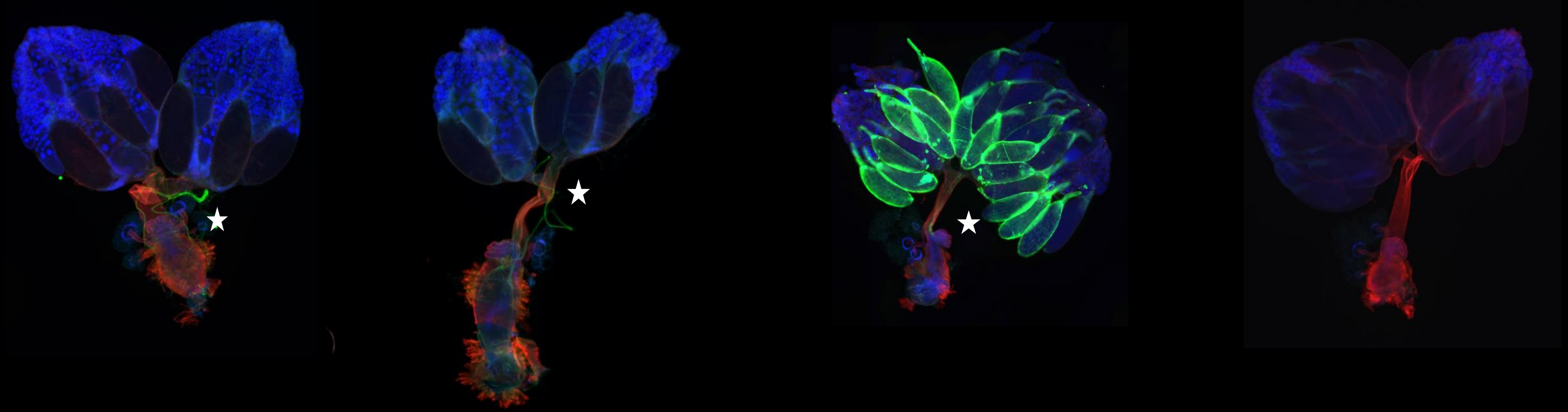

DAPI
GFP
Phalloidin

★ denotes reporter expression
