# Supplementary material for: GAL4-based functional screen of neuropeptides in Drosophila reproduction
Source: PLoS One. 2026 Mar 27;21(3):e0345918. doi: 10.1371/journal.pone.0345918 (PMC13028521; doi:10.1371/journal.pone.0345918)
Supplement: Attachment — Submitted filename: Review_PONE-D-25-58401.pdf [file pone.0345918.s003.pdf]

Article Review Submission PONE-D-25-58401

In the article entitled ”GAL4-based functional screen of neuropeptides in Drosophila reproduction”, the authors investigated the potential role of 25 neuropeptides in reproduction. They assessed the number of eggs laid after disrupting the targeted neuropeptides and examined on parallel their expression in the brain and ovary.

The article is well-written, the experiment is well-designed and the conclusions are supported by the data. The two figures and three tables chosen to illustrate the data are relevant. However, I suggest modifying Figure 1, which lacks precisions for non-experts, and Table 3, which contains redundancies with Figure 2. In addition, the article could benefit from additional details to help non-experts better understand the strategy. Below are some comments to consider in order to improve the manuscript. I suggest reorganizing a little bit the discussion and shortening it by about half, since it is long and very descriptive, and most of the neuropeptide descriptions are actually already detailed in Table 2. Most of my remarks may be naive but I think that including some modifications would make the article accessible to a wider audience.

Abstract

Line 33. I am not sure to understand the meaning of the term “readout”. Would the term “trait” be more appropriate?

Line 34. Replace implicated by “described or known to have a role”

Introduction

Line 82. Here, I think you could explain the strategy and refer to Figure 1, which could include additional details. It was unclear to me how the constructs were obtained, and what role each of them played in the study. Since I am not familiar with the field, I tried to find more information in the references listed in the text but that was time-consuming.

Could you improve Figure 1 taking some good ideas from the following scheme?

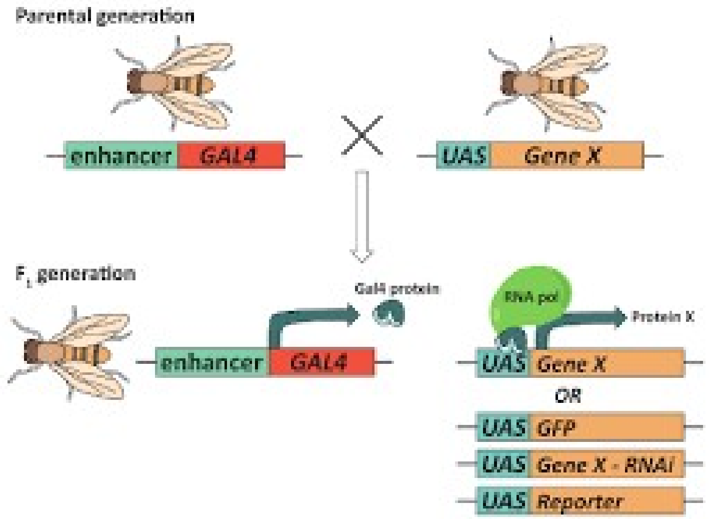

In your experiment, is Gal4 associated with gene X (neuropeptide) and UAS either with GFP (expression) or tetanus toxin (disruption of targeted gene?). It is unclear to me how this toxin works.

I suggest referencing the figure 1 here in the introduction part.

Material and methods

Line 103. Table 1. Reference to Table 1 is only mentioned in this section. However, you do not explain why sometimes you used 1, 2 or three genotypes to one neuropeptides. Please explain the relevance. Do you think it is worth discussing some of your results concerning some genotypes where the gal-transgene is inserted in the X chromosome?

Line 108. What is the mouse anti-bruchpilot for?

Line 122. Modify the sentence as follows (not sure)?
Files were kept for 6 days (6-day-old flies of 6DO flies if you want to use this abbreviation in the text). I am not sure about the expression "Flies were aged for 6 days".
Line 124. For my personal curiosity, please explain why flies were anesthetized with CO2 (in the air ? what %). It is to facilitate their manipulation and distribution into experimental groups?
Throughout the text, maybe replace egg-laying by "number of laid eggs". The term "egg-laying" is vaguer and suggests the capacity/ability of laying eggs. For my information: Is every egg laid necessarily fertilized? Would it have been interesting to let the flies hatch to verify that they are viable?
Line 131. Replace "date analysis" by data analysis
Line 137. Table 3 is cited before Table 2. I suggest removing the sentence. Sample size (n)… Or replace by "depending on the genotype, 5 to 31 individuals were used for assessing egg numbers". The same for expression? If not please indicate.

Results and discussion.

Main comment:
This section is well described and very informative but I think it could benefit for shortening. Indeed, the table 2 already gives an overview of their respective role. The number of references is very high (252).

Line 148. You wrote that NPF was excluded from the current screen but I can see sNPF in the table 3. Is it the same as NPF? Was it used as a potential positive control? If so, how do you explain that there is no statistical difference in the number of eggs with or without gene disruption?

Lane 191. You might explain a little bit more about the expression, the number of genes that are expressed in both the brain and the ovary, in the brain, and the ovary only etc. What would you expect from an expression by the brain and the ovary? or only in the brain or in the ovary? I think you should also insist on some expressional discrepancies depending on the genotypes (ex burs, CAPA, or PDF). Is it consistent with available literature?

Egg-laying results.
Line 205.
To shorten the "results-discussion" section, one suggestion is to remove any discussion of peptides whose effect are inconclusive.

Conclusions.
The paragraphs relating to limitations are very valuable and demonstrate the author's insight.

References.
I suggest reducing the number of publications cited and promoting those that are open access. I have not checked all of them but looking for information on the GAL4/UAS strategy, I realized that the reference 21 was not an open access one.

Table 3. Please indicate the meaning of n/a.
Figure 2D. Please include bars with ns for comparisons with CS (like in the other graphs).
